# Genomic modules and intramodular network concordance in susceptible and resilient male mice across models of stress

**DOI:** 10.1038/s41386-021-01219-8

**Published:** 2021-11-30

**Authors:** Salvatore G. Caradonna, Tie-Yuan Zhang, Nicholas O’Toole, Mo-Jun Shen, Huzefa Khalil, Nathan R. Einhorn, Xianglan Wen, Carine Parent, Francis S. Lee, Huda Akil, Michael J. Meaney, Bruce S. McEwen, Jordan Marrocco

**Affiliations:** 1 Laboratory of Neuroendocrinology, The Rockefeller University, New York, NY USA; 2 Douglas Mental Health University Institute, McGill University, Montreal, QC Canada; 3 Singapore Institute for Clinical Sciences, Singapore, Singapore; 4 Michigan Neuroscience Institute, University of Michigan, Ann Arbor, MI USA; 5 Department of Psychiatry, Sackler Institute for Developmental Psychobiology, Weill Cornell Medical College, New York, NY USA; 6 Yong Loo Lin School of Medicine, Singapore, Singapore; 7 Sackler Program for Epigenetics & Psychobiology, McGill University, Montreal, QC Canada

**Keywords:** Stress and resilience, Predictive markers

## Abstract

The multifactorial etiology of stress-related disorders necessitates a constant interrogation of the molecular convergences in preclinical models of stress that use disparate paradigms as stressors spanning from environmental challenges to genetic predisposition to hormonal signaling. Using RNA-sequencing, we investigated the genomic signatures in the ventral hippocampus common to mouse models of stress. Chronic oral corticosterone (CORT) induced increased anxiety- and depression-like behavior in wild-type male mice and male mice heterozygous for the gene coding for brain-derived neurotrophic factor Val66Met, a variant associated with genetic susceptibility to stress. In a separate set of male mice, chronic social defeat stress (CSDS) led to a susceptible or a resilient population, whose proportion was dependent on housing conditions, namely standard housing or enriched environment. Rank-rank-hypergeometric overlap (RRHO), a threshold-free approach that ranks genes by their *p* value and effect size direction, was used to identify genes from a continuous gradient of significancy that were concordant across groups. In mice treated with CORT and in standard-housed susceptible mice, differentially expressed genes (DEGs) were concordant for gene networks involved in neurotransmission, cytoskeleton function, and vascularization. Weighted gene co-expression analysis generated 54 gene hub modules and revealed two modules in which both CORT and CSDS-induced enrichment in DEGs, whose function was concordant with the RRHO predictions, and correlated with behavioral resilience or susceptibility. These data showed transcriptional concordance across models in which the stress coping depends upon hormonal, environmental, or genetic factors revealing common genomic drivers that embody the multifaceted nature of stress-related disorders.

## Introduction

Stressors induce a variety of molecular and physiological mechanisms, influenced by genes and environment, leading to allostatic load and alterations in coping mechanisms [1, 2].

The hippocampal formation, involved in the activation and feedback of the stress response [3–7], includes the ventral hippocampus (vHPC), a functionally discrete structure [8, 9] encoding affective behavior, that is anxiety [10], depression [11, 12], and social avoidance [13]. The vHPC expresses patterns of genetic [14, 15], and epigenetic [5, 16] regulation that differ from the dorsal hippocampus. Notably, clinical evidence shows that the vHPC, or the anterior hippocampal portion in humans, also modulates affective processes [8] associated with changes in brain volume, cell type [17], and gene expression [18]. Recently, we showed that stress induces greater alteration of gene expression change in the vHPC as opposed to the dorsal hippocampus [19] and others have characterized gene networks in the vHPC using disparate paradigms of stress [20–22]. However, genomic commonalities in the vHPC across multiple models of stress are largely uncharacterized.

Whole-genome RNA-sequencing (RNA-seq) combined with weighted gene co-expression network analysis (WGCNA) [23] was used to examine transcriptomic signatures across animal models using disparate stressors. Corticosterone (CORT) in drinking water, a noninvasive pharmacological model to deliver CORT, induces behavioral impairment in wild-type male mice [24] and disrupts the activity of the HPA axis, leading to a blunted endocrine response to stress [24]. Oral CORT delivery in male mice induces hyperphagia, locomotion [24], and stress-induced grooming, rearing, and exploratory behavior [25, 26]. Others have shown that oral CORT induces decreased prepulse inhibition in male mice heterozygous for the gene coding brain-derived neurotrophic factor Val66Met (BDNF het-Met) [27], a variant that disrupts intracellular trafficking and activity-dependent release of BDNF, modulates neurotransmitter release [28], long term potentiation [29], and results in increased risk for stress-inducible pathologies [30, 31]. Of note, BDNF het-Met male mice show increased HPA axis activity as well as increased affective behavior [32], but only after sub-chronic stress [27], indicating that an environmental challenge is needed to exhibit behavioral impairment. BDNF het-Met mice were then used to investigate the behavioral and genomic effects of oral CORT to study genetic vulnerability in a model that exhibits both an impaired stress response and altered stress-dependent affective behavior. In a separate model, chronic social defeat stress (CSDS) in adulthood, a naturalistic chronic stress model that reflects uncontrollable social aggression [33, 34], was used to distinguish a behaviorally resilient (RES) and susceptible (SUS) population of male mice [35]. Mice subjected to CSDS were raised in either standard housing (SH) or enriched environment (EE), which introduced an additional environmental component to stress susceptibility [36, 37].

Here, the pharmacological/genetic (CORT) and environmental (housing, CSDS) effects on behavior and genomics were isolated in either model, and WGCNA was used to combine bioinformatic predictions across models, revealing two networks of converging gene hubs in groups exhibiting either behavioral susceptibility or behavioral resilience.

## Methods

### Animals

**Fig. 1:**
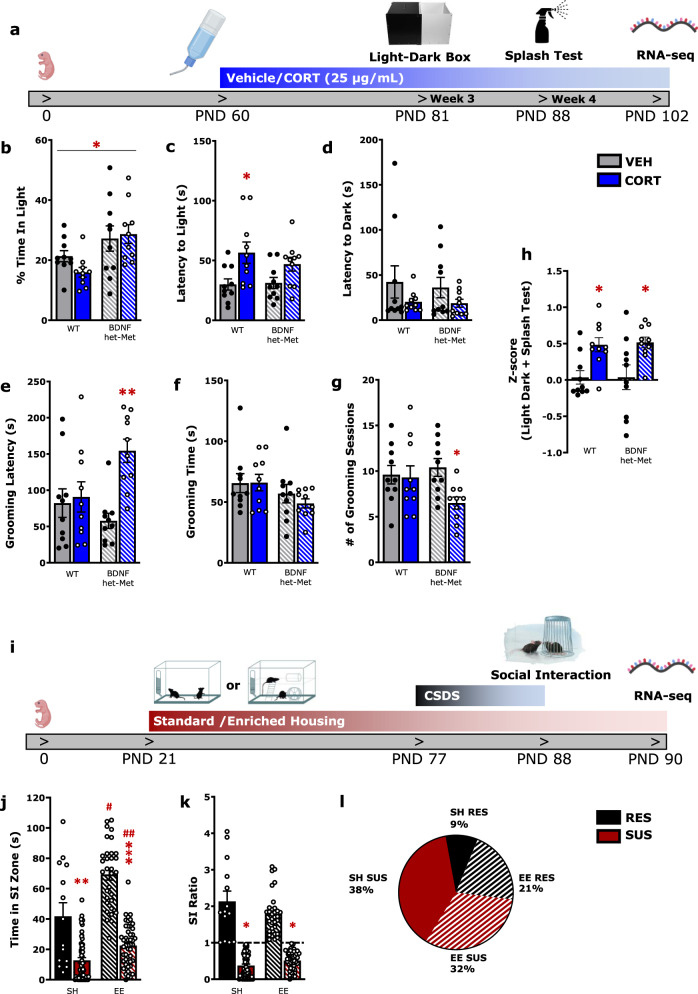
Measurements of emotional behavior in the CORT model and CSDS model show similar face validity in male mice. **a** Timeline for CORT treatment experiment. **b**–**d** Light–dark box test reveals increased anxiety-like behavior in mice treated with CORT. (2-way ANOVA, % Time in Light: genotype: *F* (1, 36) = 10.36, *p* < 0.01; Latency to Light: treatment: *F* (1, 36) = 11.47, *p* < 0.01). **e**–**g** Splash test reveals increased anxiety-like behavior in BDNF het-Met mice treated with CORT (2-way ANOVA, grooming latency: treatment × genotype: *F* (1, 36) = 6.523, *p* < 0.05; # of grooming sessions: treatment: *F*(1, 36) = 4.39, *p* < 0.05). **h** Complementary variables of behavior for both WT and BDNF het-Met were compiled to calculate a z-score, which show that CORT-treated mice displayed higher emotionality scores compared to vehicle-treated mice regardless of genotype (2-way ANOVA, treatment: *F* (1, 36) = 16.44, *p* < 0.001). **i** Timeline for CSDS treatment experiment. **j**, **k** The social interaction test reveals decreased anxiety-like behavior in RES mice. (2-way ANOVA, Time in SI Zone: interaction (susceptibility-by-housing) effect: *F* (1, 150) = 7.068, *p* < 0.01; SI Ratio: susceptibility effect: *F* (1, 150) = 243.2, *p* < 0.001). **l** Pie-chart depicting the proportion of mice raised in SH or EE and ranked RES or SUS after CSDS. The proportion of RES mice raised in EE (21%) was higher than the one of RES mice raised in SH (9%) (*p* < 0.05). Columns represent the mean ± S.E.M. of ten animals per group for the CORT model (**a**–**h**) and mean ± S.E.M. of 59 animals for SUS and 14 for RES in SH, as well as 49 animals for SUS and 32 for RES in EE (**j**, **k**). ^*/#^*p* < 0.05; **p < 0.01; ^***/##^*p* < 0.001. PND post-natal day, WT wild-type, BDNF het-Met heterozygous BDNF Val66Met, VEH vehicle, CORT corticosterone, CSDS chronic social defeat stress, SI social interaction, RES resilient, SUS susceptible, SH standard housing, and EE enriched environment.

Mice heterozygous for the BDNF allele (het-Met) were generated in the Lee laboratory, as previously described [30]. C57/BL6J male mice (WT) and BDNF het-Met mice were obtained by performing in-house breeding. A separate cohort of C57/BL6J male mice (21 day old) was randomly assigned housing in either SH or EE conditions for 8 weeks. At 2 months of age WT or BDNF het-Met mice were randomly assigned to either vehicle- (1% ethanol) or CORT- (25 mg/l, 1% ethanol) treated groups. At 3 and 4 weeks into the treatment course, mice were tested using the light–dark box test and splash test, respectively (see Supplementary Materials) (Fig. 1a). After the 8 weeks in their respective housing, the EE and SH cohort underwent 10 days of CSDS, followed by the social interaction (SI) test (see Supplementary Materials) (Fig. 1i). All procedures were performed in accordance with the National Guidelines on the Care and Use of Animals and a protocol approved by The Rockefeller University Animal Care and Use Committee and the Canadian Council on Animal Care with protocols approved by the McGill University Facility Animal Care Committee.

### RNA-seq

After 6-week-CORT treatment, mice were killed by cervical dislocation. The vHPC was dissected, immediately flash frozen, and stored at −80 °C. The vHPC is enriched in glucocorticoid receptors, particularly in the dentate gyrus [38]. The ventral dentate gyrus was isolated at P90 from mice that underwent CSDS, and the tissues were rapidly removed, flash frozen, and stored at −80 °C. In CORT-treated mice, three biological replicates per experimental group were processed, with each replicate comprising of RNA pooled from two animals (see Supplementary Materials) for sequencing. One to seven replicates of RNA sample (not pooled) from each CSDS experimental group of animals were randomly selected for the RNA library preparation. Quality control was performed on the reads obtained from the core and reads with a score of <15 were discarded [39, 40]. The reads were then aligned to the GRCm38 genome using the STAR aligner [41] with Ensembl annotation [42] and quantified to the gene level using featureCounts [43]. The read counts were analyzed using the R/Bioconductor framework [44] (www.R-project.org).

### Sequencing analysis and statistics

Behavioral data were analyzed using GraphPad Prism (San Diego, CA, USA) by performing a two-way ANOVA followed by Newman–Keuls post hoc analysis for multiple comparisons. A *p* value < 0.05 was set for statistical significance. Z-score was used to compile complementary variables of behavior (see Supplementary Materials). Differentially expressed genes (DEGs) were obtained using the Limma–Voom package [45] (uncorrected *p* < 0.05, fold change > 1.3). Overlaps between the differential expression of two independent RNA-seq comparisons were analyzed with the “stratified” rank-rank hypergeometric overlap (RRHO) analysis [46]. The raw reads were independently processed through WGCNA, a systems biology analysis method for describing the correlation pattern among genes across samples [47]. Enrichment of DEGs within each WGCNA module was assessed through Fisher’s exact test corrected for multiple testing (Benjamini-Hochberg False Discovery Rate < 0.05) with fold change > 1.3. Gene ontology (GO) categories were manually curated from results of the Database for Annotation, Visualization and Integrated Discovery functional annotation cluster tool, where the top five pathways with higher enrichment score were selected using the gene sets generated from RRHO overlaps with concordant gene networks.

## Results

### Behavioral phenotyping of male mice under CORT treatment or CSDS combined with alternate housing

Previous reports show that low dose oral CORT does not induce significant body change compared to vehicle [24] (Supplementary Table 1). Similarly, 10 days of CSDS do not lead to body weight differences compared to unstressed mice [33, 48, 49], however SUS mice show increased body weight compared to RES mice [33] unless raised in EE [50]. The cohort of mice maintained on CORT treatment was assessed for anxiety- and depression-like behavior using the light–dark box test and the splash test, two behavioral paradigms used for the first time here in mice treated with chronic oral CORT. At day 21 of treatment, mice were tested in the light–dark box. WT mice, regardless of treatment, spent less time in the light box when compared to BDNF het-Met mice (Fig. 1b). In addition, CORT induced increased latency to enter the light box in WT mice but not in BDNF het-Met mice compared to their respective vehicle-treated mice (Fig. 1c). No differences were found in the latency to enter the dark box in either treatment or genotype (Fig. 1d). Mice were maintained on oral CORT, and 1 week later they were tested using the splash test, where reduced grooming meets face validity for increased depression-like behavior [51]. Here, CORT induced depression-like behavior, although this effect was limited to BDNF het-Met mice when testing grooming latency and the number of grooming sessions compared to their controls treated with vehicle (Fig. 1e, g). No differences were found in the time spent grooming across groups (Fig. 1f). Thus, WT and BDNF het-Met mice displayed specificity to either test for anxiety-like behavior and depression-like behavior, respectively. To increase the sensitivity and reliability of the behavioral measurements, we applied z-normalization across complementary variables scored in both tests [52]. The distribution of the z-scores showed a cumulative increased anxiety- and depression-like behavior in CORT-treated mice compared to vehicle-treated mice regardless of genotype (Fig. 1h).

A separate set of mice housed in either SH or EE underwent CSDS and was then assessed for social behavior using the social interaction (SI) test. SUS mice exhibited CSDS-induced reduction in time spent in the SI zone when the CD1 aggressor was present (Fig. 1j). Mice that scored an SI ratio greater than 1 were classified as RES, whereas mice scoring lower than 1 were classified as SUS to CSDS [33] (Fig. 1k). In both RES and SUS mice, EE induced increased time spent in the SI zone compared to mice raised in SH (Fig. 1j). The proportion of mice ranked as either SUS or RES considerably changed when mice were raised in SH or EE. Indeed, of the total cohort of mice, 21% were RES and raised in EE compared to only 9% of the cohort ranking RES after SH (Fig. 1l).

Together, these results indicate that chronic treatment with oral CORT or CSDS after alternate housing led to a distinct set of behavioral phenotypes that converged towards increased affective behavior, namely the light–dark box test and splash test for anxiety-like and depression-like behavior and SI test for impaired social behavior.

### Converging transcriptional responses of oral CORT and CSDS

We next investigated the genomic correlates of the behavioral phenotypes observed in CORT-treated mice and mice exposed to CSDS that were housed in either SH or EE and examined whether there were converging transcriptional responses. The behavioral findings favored the genomic focus in the vHPC, whose pharmacological modulation meets face validity for the behavioral paradigms reported in this study [6, 10, 53–56]. RNA-seq was used to identify transcriptional synchrony in DEGs across groups. CORT induced 220 DEGs in WT and 569 DEGs in BDNF het-Met mice compared to vehicle, with only 62 common DEGs across genotypes. CSDS-induced 273 and 238 DEGs between SUS mice compared to RES mice in animals raised in SH and EE, respectively, sharing only 15 common DEGs across housing conditions (Supplementary Data 1). The number of DEGs across comparisons indicated that CORT and CSDS-induced gene expression change in the vHPC as a function of genotype and housing.

**Fig. 2:**
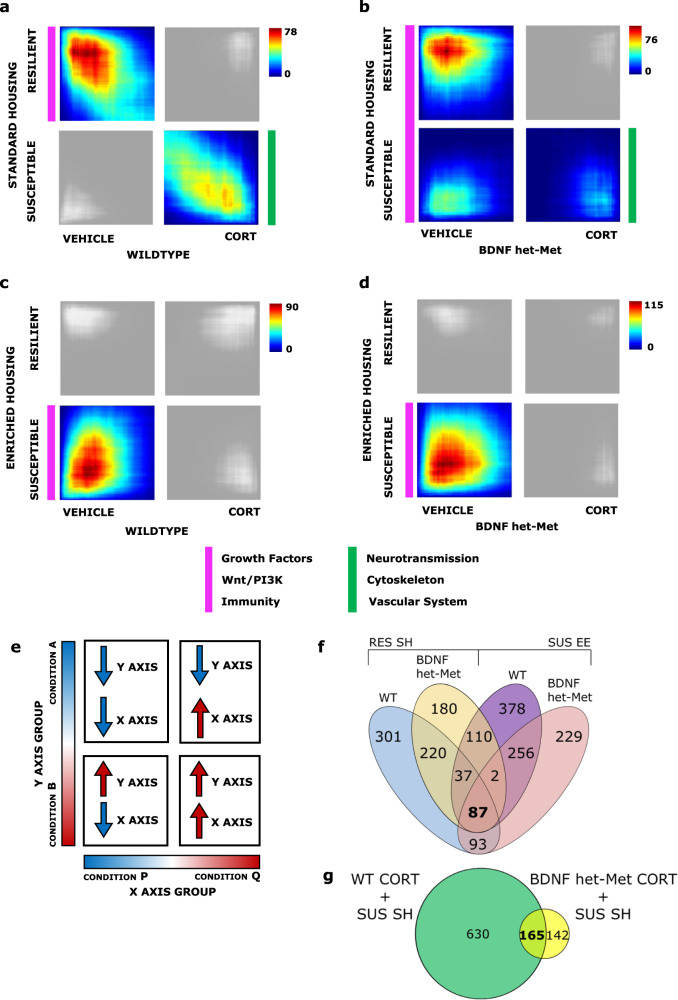
Genomic signatures parallels vulnerability to stress across models and is modulated by housing condition or genotype. **a**–**d** Each RRHO contained 17,122 DEGs across the four quadrants. Pixels represent the overlap between the transcriptome of each comparison as noted, with the significance of overlap [−log 10 (*p* value) of a hypergeometric test] color coded. Quadrants that were not significant to the analysis are shaded in gray. **a** Comparison of SUS versus RES DEGs in SH with vehicle versus CORT DEGs in WT (max −log 10 (*p* value) = 78); RES and vehicle: 738 DEGs; SUS and CORT: 795 DEGs. **b** Comparison of SUS versus RES DEGs in SH with vehicle versus CORT DEGs in BDNF het-Met (max −log 10 (*p* value) = 76); RES and vehicle: 636 DEGs; SUS and vehicle 522; SUS and CORT: 307 DEGs. **c** Comparison of SUS versus RES DEGs in EE with vehicle versus CORT DEGs in WT (max −log 10 (*p* value) = 90); SUS and vehicle: 667 DEGs. **d** Comparison of SUS versus RES DEGs in EE with vehicle versus CORT DEGs in BDNF het-Met (max −log 10 (*p* value) = 115); SUS and vehicle: 870 DEGs. Parallel colored markers indicate macro modules with converging GO terms across quadrants. **e** Representative image of the following threshold-free rank-rank hypergeometric overlap comparisons of DEGs. The direction of regulation in each quadrant that corresponds to each variable on each axis is described with an up or down arrow. The upper left quadrant designates co-downregulated genes, lower right quadrant designates co-upregulated genes, and the lower left and upper right quadrants include oppositely regulated genes (up-down and down-up, respectively). Genes along each axis are listed from greatest to least significantly regulated from the outer to middle corners. Genes are listed in Supplementary Table 1. Venn diagrams showed the common genes between experimental groups of the RRHO quadrants depicted in Fig. 2 corresponding to the point of highest overlap of each quadrant as described in Plaiser et al. [57] (see Supplementary Methods). **f** RES mice in SH and either WT mice (blue) or BDNF het-Met mice under vehicle (yellow), and between SUS in EE and either WT mice (purple) or BDNF het-Met mice under vehicle (coral), with 87 genes (Supplementary Data 2) shared across all comparisons (salmon); **g** SUS mice in SH and either WT (green) or BDNF het-Met mice (yellow) under CORT, with 165 (Supplementary Data 2) shared across both comparisons (lime). RRHO rank-rank hypergeometric overlap, DEG differentially expressed gene, WT wild-type, BDNF het-Met heterozygous BDNF Val66Met, CORT corticosterone, SI social interaction, RES resilient, SUS susceptible, SH standard housing, EE enriched environment, and GO gene ontology.

Stratified RRHO test [46], a threshold-free approach that ranks genes by their *p* value and effect size direction, combined with GO was used to identify genes from a continuous gradient of significancy that were concordant across models and the biological pathways that corresponded to common DEGs of the highest rank between models. Significant overlaps were identified using the point of highest −log 10 (*p* value) from each quadrant as described in Plaiser et al. [57] (see  Supplementary Methods) with nonrelevant quadrants shaded in gray. We identified a robust overlap between genes downregulated in WT mice under CORT compared to vehicle-treated mice and downregulated in SUS mice compared to RES mice both raised in SH. We also observed a significant overlap between upregulated genes in WT mice under CORT compared to vehicle and SUS mice compared to RES mice raised in SH (Fig. 2a). The same patterns of overlap, albeit smaller, were also observed when in the analysis WT mice were replaced with BDNF het-Met mice treated with CORT (Fig. 2b). We also found a significant overlap in DEGs upregulated in WT mice under vehicle and SUS mice raised in EE, compared to CORT and RES mice, respectively (Fig. 2c). This same pattern of overlap was observed in BDNF het-Met mice treated with vehicle (Fig. 2d). DEGs from each overlap were grouped according to their GO, which showed two main macro-networks of genes that converged in discrete comparisons (Supplementary Table 2). The network induced in SUS mice raised in SH and mice treated with CORT of both genotypes was implicated in the regulation of neurotransmission, cytoskeleton function, and brain vascularization, especially regarding oxygen transport and iron binding (Supplementary Table 2; Supplementary Data 2). Specifically, CORT-treated WT and BDNF het-Met mice shared 165 genes with SUS mice raised in SH (Fig. 2g; Supplementary Data 2). In the network that included genes concordant in RES mice raised in SH and vehicle-treated mice of both genotypes, GO terms mainly referred to the immune response, the Wnt/PI3K pathway activation, or coded for growth factors (Supplementary Table 2; Supplementary Data 2). Curiously, about 40% of DEGs with these same functions were also expressed in SUS mice raised in EE and vehicle-treated mice regardless of genotype (Supplementary Data 2). Thus, SUS mice raised in EE expressed a novel set of genes that shifted the overlap away from CORT-treated mice. We identified 87 genes that were common in RES mice raised in SH and SUS mice raised in EE and that also matched with vehicle-treated mice regardless of genotype (Fig. 2f; Supplementary Data 2). A different set of genes that was also involved in neurotransmission, cytoskeleton function, and brain vascularization was common to BDNF het-Met mice under vehicle and SUS mice raised in SH, suggesting that, as opposed to WT, the BDNF Met variant alone moved the DEGs convergence towards SUS mice raised in SH.

Together, we showed that CSDS in SUS mice raised in SH induced gene networks common to mice treated with CORT, but EE in SUS mice dramatically reduced this similarity.

### Selection of multimodel gene networks based on co-expression analysis

**Fig. 3:**
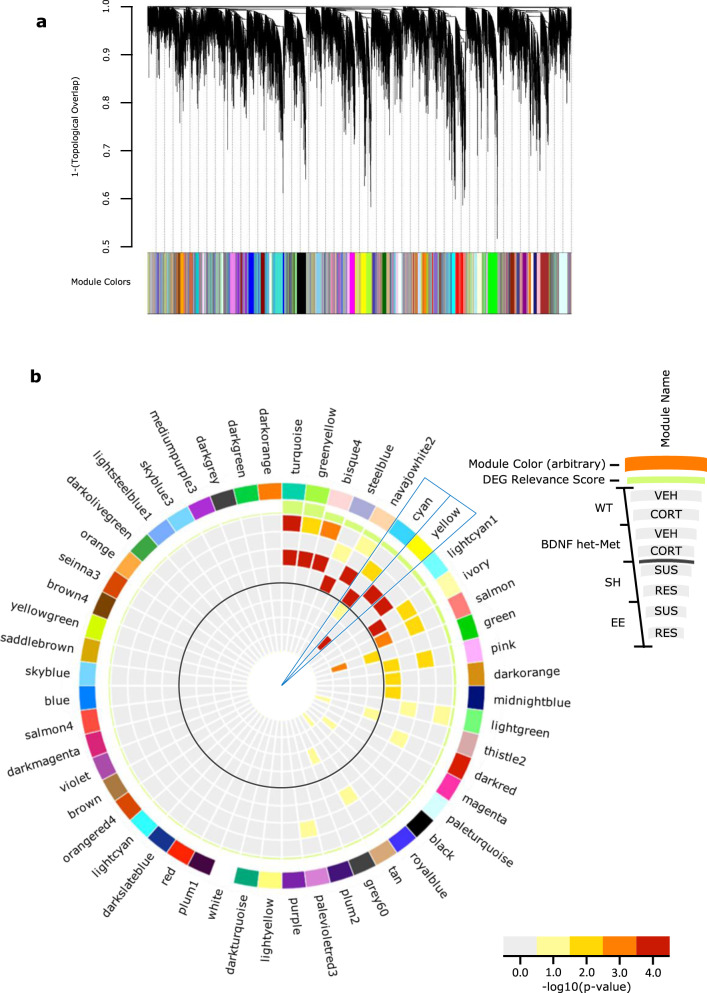
Identification of shared-model co-expression networks and key modules. **a** Consensus co-expression network analysis identified 54 coexpressed modules in both the CORT and CSDS experiments via hierarchal gene clustering on TOM-based dissimilarity and branch cutting using the top-down dynamic tree cut method. Each module is assigned a unique color identifier along the bottom of the dendrogram. Dendrograms demonstrate average linkage hierarchical clustering of genes based on the calculated topological overlap values. **b** Circos plot showing module name (ring 1), color (ring 2), a differential expression relevance score; calculated from the average enrichment of DEGs across all groups, with increasing bar height indicating increased average total enrichment (ring 3). Bar color indicates the significance for genes upregulated for each condition (vehicle compared to CORT, CORT compared to vehicle, SUS compared to RES, and RES compared to SUS) with warmer colors signifying increasing −log 10 (*p* value). The solid gray circle indicates the separation between the DEGs of the model of oral CORT in the outer shell and the DEGs of the model of differential housing before CSDS in the inner shell. The *cyan* and *yellow* modules are indicated as they have higher DEG average relevance, along with significant enrichment between models. WT VEH up (ring 4), WT CORT up (ring 5), BDNF het-Met VEH up (ring 6), BDNF het-Met CORT up (ring 7), SH SUS up (ring 8), SH RES up (ring 9), EE SUS up (ring 10), and EE RES up (ring 11). CORT corticosterone, CSDS chronic social defeat stress, TOM topological overlap matrix, DEG differentially expressed gene, WT wild-type, BDNF het-Met heterozygous BDNF Val66Met, VEH vehicle, RES resilient, SUS susceptible, SH standard housing, and EE enriched environment.

Moving from the RRHOs predictions, we sought to investigate whether the gene network converging groups met significancy for gene co-expression. Gene co-expression analysis is useful in identifying transcriptional alterations in genetically complex disorders, where the phenotype is a consequence of numerous small genomic alterations rather than from isolated single-gene effects [58]. Using weighted gene co-expression analysis (WGCNA), we constructed a consensus gene co-expression network and identified 54 gene modules that differ in topological overlap, each of which included a unique network of interconnected gene hubs (Fig. 3) based on hierarchal clustering and network preservation (Supplementary Fig. 1) [23]. Each module was assigned an arbitrary color, and we found that the *cyan* and *yellow* module were the only two modules that showed enrichment of DEGs across the combination of all experimental groups (Fig. 3b). The *cyan* module included upregulated DEGs of mice under CORT of both genotypes and SUS mice raised in SH, compared to vehicle-treated mice and RES mice, respectively. The *yellow* module included DEGs that were upregulated in BDNF het-Met mice under vehicle and SUS mice in EE, compared to mice under CORT and RES mice, respectively. This pattern of enrichment was consistent with the overlaps observed in the RRHO analysis (Fig. 2a, b, d). Also, the *cyan* module included groups that showed increased affective behavior (Fig. 1b, c, e, g, h, j, k), while the *yellow* module included groups that exhibited decreased affective behavior (Fig. 1e, g, h, j–l), with gene hubs that were exclusive to either module.

### Analyzing the intramodular network structure and function of key modules

**Fig. 4:**
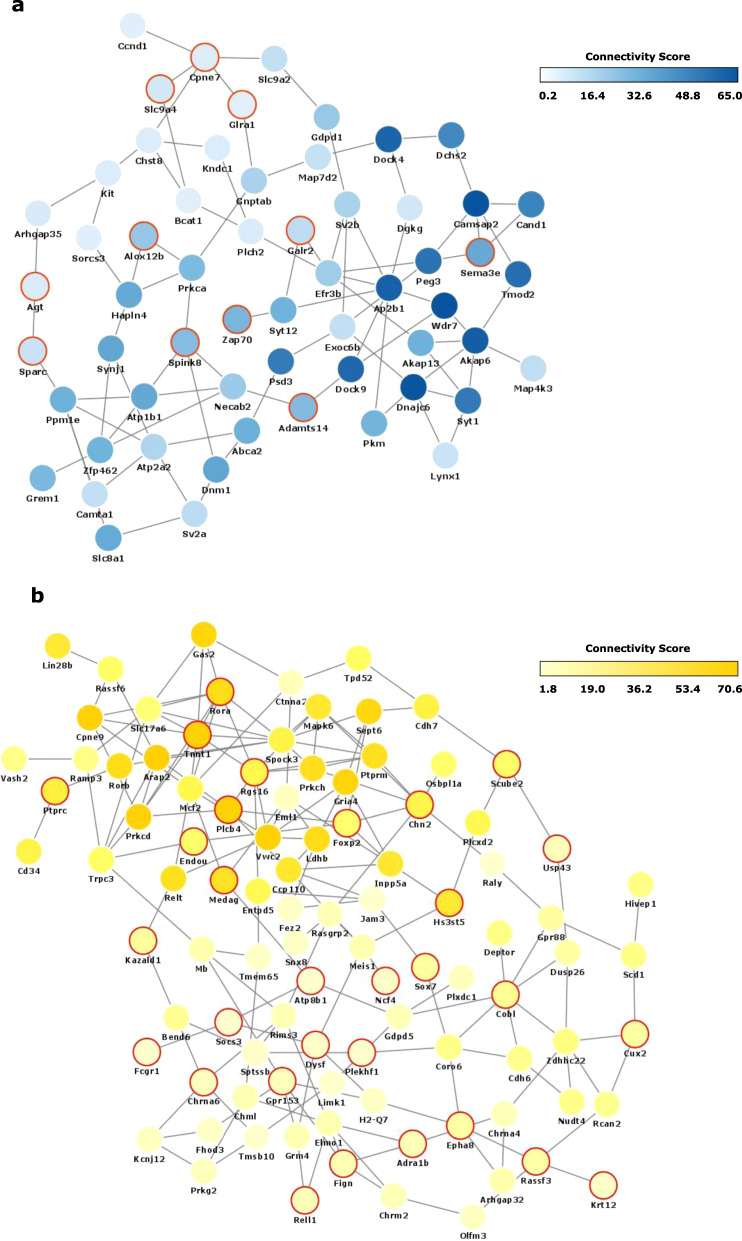
Co-expression networks of *Yellow* and *Cyan* modules. **a** Network plot of hub genes within the *cyan* module. **b** Network plot of hub genes within the *yellow* module. Most significant nodes were selected from the most connected genes >1 standard deviation from the average (μ + σ) when organized by decreasing kME score. Node color is proportional to the node’s level of connectivity within the hub. Red outline indicates a differentially expressed gene.

To identify drivers of the target modules, we reconstructed the network of genes within each module based on their co-expression-based interconnectivity and then identified “hub genes” (Fig. 4a, b). Hub genes are highly connected genes within a module that are expected to control the expression of other module members. The three highest connected hub genes in the *cyan* module included *Wdr7*, *Camsap2*, and *Dnajc6* (Supplementary Data 3). *Cyan* hub genes that were differentially expressed and shared across groups were *Spink8* and *Krt73*, upregulated in both WT and BDNF het-Met mice under CORT, *Alox12b*, upregulated in both WT mice under CORT and SUS mice raised in SH, and *Adamts14*, upregulated in both BDNF het-Met mice under CORT and SUS mice raised in SH (Supplementary Table 3; Supplementary Data 3). The three highest connected hub genes in the *yellow* module included *Arap2*, *Vwc2*, and *Cpne9* (Supplementary Data 3). In the *yellow* module, *Usp43* was the only gene differentially expressed in both BDNF het-Met mice under vehicle and SUS in EE (Supplementary Table 3; Data 3). Other *yellow* hub genes that were differentially expressed and showed high connectivity score included *Plcβ4*, upregulated in SUS mice raised in EE, and *Tnnt1* and *Chn2*, upregulated in BDNF het-Met mice under CORT (Supplementary Table 3). Together, the hub genes showed that there existed key drivers exclusive to either the *yellow* or the *cyan* module in groups showing decreased or increased affective behavior, respectively.

## Discussion

We investigated functional gene networks in animal models of stress with different construct validity, i.e., distinct stressors, by conducting co-expression analysis of DEGs. Unique hub genes and co-expression networks were exclusive to mice exhibiting either behavioral susceptibility or resilience to stress.

CORT induced increased affective behavior in both WT and BDNF het-Met male mice, indicating that both genotypes showed behavioral susceptibility to CORT as others have also reported [25, 59, 60]. These findings were validated by compiling complementary behavioral variables that meet converging face validity [61, 62], a translational application that recapitulates illness definition (i.e., a syndrome as a collection of variable symptoms) by incorporating converging variables of symptoms, especially in affective disorders [52]. Anxiety- and depression-like behavior were studied here for the first time in oral CORT-treated mice using classical paradigms such as the light–dark box test or the splash test, which do not entail prior stressors. Behavioral susceptibility to CSDS [63] varied significantly whether mice were raised in SH or EE and was consistent with findings showing that EE reduces emotional lability [64] even when experienced prior to stress [65]. The SI test analyzes a unique trait of depression-like behavior and shows pharmacological validity for stress-related disorders [66].

To determine whether there existed converging gene networks in the brain that drove the observed stress-related phenotypes, we focused on the vHPC, a brain region whose pharmacological and anatomical manipulation has been shown to induce effects in the same behavioral tests used in this study [10, 53–56]. Also, CORT treatment and environmental enrichment have been associated with selective changes in neurogenesis [67] and gene expression [5] in the vHPC. Others have reported stress-induced gene expression differences across distinct brain regions, such as the nucleus accumbens or the medial prefrontal cortex, especially after CSDS [3, 68, 69]. It remains to investigate whether other brain regions would also show converging genomic signatures across the paradigms reported here. Interestingly, when comparing our dataset with studies using different stressors, such as CSDS [70] and unpredictable chronic mild stress [71], we found that some DEGs were also differentially regulated across other models and brain regions, including the hippocampus, amygdala, prefrontal cortex, and nucleus accumbens (Supplementary Table 4). Cross-model, cross-tissue genomic commonalities shows that several hub genes observed in mice treated with CORT or raised in EE or SH prior exposure to CSDS have validity beyond the stressors used in this study.

By referencing novel RNA-seq data from the vHPC, we used RRHO combined with GO to depict the multimodel trends of transcriptional regulation and biological function. One macro network, which included genes that participates in the immune response, Wnt/PI3K pathway activation, or coding for growth factors was found in quadrants associated with behavioral resilience, while a distinct macro network implicated in neurotransmission, cytoskeleton function, and brain vascularization, was expressed in quadrants associated with behavioral susceptibility. The immune system is influenced by stress and glucocorticoids [72, 73], and along with Wnt signaling and growth factors has been implicated in the pathogenesis of depression [72–74]. Furthermore, disruption of cytoskeleton functions and alteration of brain vascularization and vessels, have also been associated with neuroanatomical changes of the hippocampus, depression-like behavior, and susceptibility to neuropsychiatric disorder [75, 76].

Biological pathways enriched in the RRHO overlaps were consistent with the distinct genomic landscape observed in stress susceptibility and resilience [3, 5]. We observed a smaller, compared to WT mice, but unique overlap between SUS mice in SH and BDNF het-Met mice treated with CORT, suggesting that exclusive sets of genes are induced by stress in BDNF het-Met mice, as others have also reported [77, 78]. The most significant overlap existed between SUS mice raised in EE and mice under vehicle of both genotypes. Thus, EE in SUS mice profoundly reduced the similarity between CORT-treated and SUS mice, shifting the overlap instead to vehicle-treated mice and indicating that EE reverses the genomic signatures of stress typically associated with susceptibility. The genomic shift observed under EE in SUS mice is consistent with findings showing that EE promotes hippocampal neurogenesis and restores behavior after social defeat [79].

**Fig. 5:**
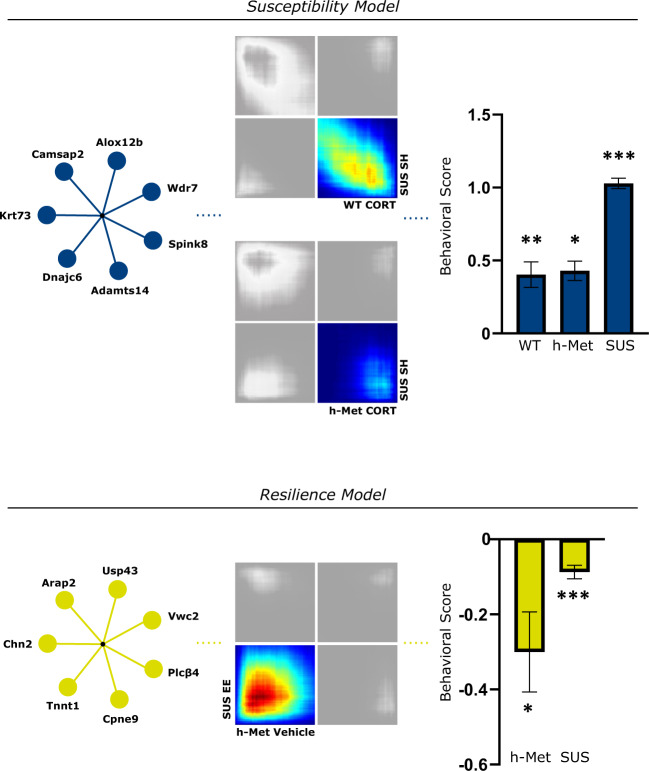
Summary of transcriptomic and behavioral synchrony. Enrichment in hub genes and DEGs in the *cyan* module, matching RRHOs, and behavioral susceptibility to stress (*cyan* column) are found in CORT-treated mice of both genotypes and SUS mice raised in SH. Enrichment in hub genes and DEGs in the *yellow* module, concordance in RRHO gene expression, and behavioral resilience to stress (*yellow* column) are found in BDNF het-Met treated with vehicle and SUS mice raised in EE. The behavioral score for each group was normalized to its respective control: WT CORT mice normalized to WT vehicle (*t* = 3.356, df = 17.95, *p* < 0.01), BDNF het-Met CORT mice normalized to BDNF het-Met vehicle (*t* = 2.579, df = 12.30, *p* < 0.05), SH SUS mice normalized to RES in SH (*t* = 5.454, df = 13.98, *p* < 0.001), BDNF het-Met vehicle mice normalized to BDNF het-Met CORT (*t* = 2.579, df = 12.30, *p* < 0.05), and EE SUS mice normalized to SUS in SH (*t* = 3.455, df = 104.6, *p* < 0.001). Behavioral score for each animal is calculated as: 1 − ${{{{{{{\mathrm{z/\bar a}}}}}}}}$, where z = [(x − x̅_cont_)/σ_cont_] * (±1)] and x = percentage time in the light, latency to dark, latency to light, grooming time, number of grooming sessions, grooming latency, time in social interaction zone, or SI ratio, and a = z for each respective control mouse. After calculation, a and z values where normalized to the lowest z score. Columns represent the mean ± S.E.M. of ten animals per group for the CORT model and mean ± S.E.M. of 59 animals for SUS in SH and 49 animals for SUS in EE. *p < 0.05; **p < 0.01; ***p < 0.001. DEG differentially expressed gene, RRHO rank-rank hypergeometric overlap, WT wild-type, BDNF het-Met/h-Met heterozygous BDNF Val66Met, CORT corticosterone, SUS susceptible, SH standard housing, and EE enriched environment.

After elucidating individual DEGs, differential connectivity analysis was used to examine multi-dimensional alterations between pairs of genes, as susceptibility to stress-related disorders is characterized by fundamental changes in the architecture of transcriptional networks [22]. WGCNA analysis showed that two modules, *yellow* and *cyan*, were enriched in DEGs across all of the experimental groups. This network-based approach allowed us to identify several hub genes, which according to their definition are more likely to drive the function of the entire network [23, 80]. The significant enrichment of the *cyan* module indicated that CORT-treated mice, regardless of genotype, exhibited gene expression synchrony with SUS mice raised in SH prior CSDS, an expression pattern that reflected increased affective behavior observed selectively in these groups. The most highly connected hub gene of the *cyan* module was *Wdr7*, whose altered expression was found in subjects with depression and alcohol dependence comorbidity [81]. The intramodular network of *Wdr7* included *Adamts14* and *Dock4*, two markers of neuropsychiatric disorder susceptibility [82, 83]. The *yellow* module demonstrated genomic synchrony in mice exhibiting low affective behavioral score, namely BDNF het-Met mice under vehicle and SUS mice raised in EE, emphasizing previous evidence showing that BDNF het-Met mice express genes associated with stress coping even in the absence of applied stressors [78]. The top hub regulator of the *yellow* module, *Arap2*, encodes for pro-survival functions, such as Akt activity [84], glucose uptake, and sphingolipid metabolism [85], and its dysregulation leads to impaired affective behavior in animal models of depression [86, 87]. In the *yellow* module, *Arap2* was interconnected with other resilience-associated genes. For example, increased expression of *Tnnt1* was observed in mice after injection with the rapid-acting antidepressant ketamine [88]. The intramodular network of *Arap2* also included *Plcβ4* and *Rora*, whose functional expression was associated with improvement in anxiety [89] and depressive [90] symptoms. Therefore, the *cyan* and *yellow* modules included hubs that associated with stress susceptibility or resilience, respectively. We offer a model that summarizes converging intramodular networks that regulate converging behavioral phenotypes, namely increased or decreased affective behavior. This scheme shows that groups included in the *cyan* module display behavioral susceptibility, while groups included in the *yellow* module exhibit behavioral resilience (Fig. 5).

Although distinct paradigms were used to study stress coping, such as genetic inheritance, hormones, and environment, there were commonalities and differences across models. One major commonality between the CSDS and CORT models is that they both show dysregulation of the HPA axis [24, 91], and both exhibit a blunted endocrine response to stress [25, 92]. Also changes in BDNF levels in mice undergoing CSDS are causally related to changes in plasma CORT and sensitivity to stress [93]. CORT treatment induces stress-related behaviors in males with negligible individual variability, that is a population resilient to oral CORT has not been reported. However, CSDS leads to the isolation of two populations that are either RES or SUS to stress [35]. Accordingly, mounting evidence shows that the intrinsic component of susceptibility in CSDS mice mostly depends on epigenetic factors and the early life environment [94, 95], while the BDNF het-Met variant confers a genetic predisposition to stress-related behavior in response to an applied stressor [32]. When comparing pharmacological and environmental models, one should consider unique physiological mechanisms due to disparate stress-inducing conditions. Nevertheless, some gene pathways may act as an independent core that may modulate the multifactorial physiological responses to stress, despite the diversity of the stressors. While this study focuses on males, data previously reported in females, from the same cohort of males included here, show that CORT reduces, rather than increases, affective behavior selectively in BDNF het-Met mice, and induces gene expression patterns that profoundly differ from those described in males [96]. In the attempt to combine models with converging face validity for behavioral susceptibility, females were not compared with CSDS mice because they showed a behavioral phenotype that opposed the one observed in males. The study of sex differences must then focus on genomic signatures underlying opposed face validity rather than converging behavioral and molecular traits. Furthermore, data in females exposed to CSDS and alternate housing are scarce, and the comparison with females treated with CORT would necessitate an alternate protocol to induce CSDS [97], thus introducing an additional variable of comparison. Notably, several hub genes found in this study are also differentially regulated in the prefrontal cortex of susceptible females undergoing CSDS [98] (Supplementary Table 4). This suggests that males and females, while showing a distinct behavioral response to CORT [96] and a distinct genomic response to stress [19, 68, 71, 78], may share a core connectome of hub genes that orchestrates the stress response. Without neglecting the importance of mosaic gene networks in complex illnesses, further studies may also secure the identification of targets at the single-gene level, using silencing/activation methods to dissect the gene driver(s) of the modular network that corroborate a causal link with the behavioral phenotypes that we reported.

Stress-related disorders are multifaceted, constraining the understanding of their molecular etiologies [99]. Investigating the holistic network of the genome associated with stress-related disorders, moving away from the one gene-one disease paradigm, could shift focus to key biological systems and facilitate the identification of novel therapeutic targets, with the scope of constructing interventions to target whole connectivity networks rather than individual DEGs [22, 100]. The distinction between the effect of different stressors on animal behavior and physiology remains largely unexplored but it has practical implications to recapitulate the complexity of human conditions in preclinical models [101], as in the case of subjects with major depressive disorders [68] (Supplementary Table 4). Using a multimodel metanalysis, this study uncovers a novel gene core of the stress response, highlighting biomarkers that underlie the susceptibility to stress-related disorders.

## Supplementary information

Supplementary Material Methods

Supplementary Figures

Supplementary Data 1

Supplementary Data 2

Supplementary Data 3

## Data Availability

RNA-seq data have been deposited to GEO GSE174664 and GSE150812. DEGs are included in Supplementary Datasets. All other data are available upon request.
