# Supplementary material for: Genomic modules and intramodular network concordance in susceptible and resilient male mice across models of stress
Source: Neuropsychopharmacology. 2021 Nov 30;47(5):987–99. doi: 10.1038/s41386-021-01219-8 (PMC8938529; doi:10.1038/s41386-021-01219-8)
Supplement: Supplementary file 2 — Supplementary Figures [file 41386_2021_1219_MOESM2_ESM.pdf]

Supplementary Table 1. Body weight over the course of CORT treatment.

	WT		BDNF het-Met	
**Week**	**Vehicle**	**CORT**	**Vehicle**	**CORT**
0	26.55 ± 0.31	25.05 ± 0.33	26.31 ± 0.49	26.92 ± 0.49
1	27.65 ± 0.31	26.19 ± 0.44	27.74 ± 0.54	27.67 ± 0.64
2	28.5 ± 0.38	27.85 ± 0.47	28.49 ± 0.54	28.77 ± 0.84
3	29.19 ± 0.42	29.75 ± 0.46	29.38 ± 0.47	29.39 ± 1.05
4	29.65 ± 0.45	30.84 ± 0.53	29.86 ± 0.50	30.06 ± 1.14
5	30.13 ± 0.52	32.09 ± 0.55	30.28 ± 0.54	31.1 ± 1.18
6	30.86 ± 0.50	32.43 ± 0.53	30.82 ± 0.55	32.12 ± 1.29

Supplementary Table 2. GO with related genes from RRHO analysis.

GO Term	Enrichment Score	# Genes	Select Genes
**Pink Module**			
Immunity	16.21	73	Fos, Ncf1, Ncf4, Iigp1, Tlr2, Tlr6, Casp8, Fcgr1, Nfkbia, H2-Aa
Extracellular Matrix	10.90	53	Cdh1, Cadm4, Itga1, Itgb2, Itgb3, Itgb4, Tgfbi, Des, Nectin2, Col2a1
Wnt/PI3K Signaling	9.70	90	Wnt3, Wnt9a, Met, Hgf, Fgfr2, Tdgf1, Fgf18, Jak3, Tek, Comp
Signal Transduction	5.11	159	Grb7, Gab1, Igfbp2, Ghsr, Fyb, Hhip, Npbwr1, Pkig, Ntsr1, Oprk1
Iron Homeostasis	2.65	11	Trf, Lcn2, Mfi2, Bdh2, Scara5, Steap1, Steap3, Steap4
**Green Module**			
Neurotransmission	11.98	64	Cacna1b, Trpm2, Slc9a5, Cacna1g, Slc38a4, Clic6, Slc9a2, Scn5a, Kcnmb2, Casr
Neuronal Differentiation	3.88	18	Shh, Otx2, Notch1, Sox5, Ar, Kdr, Insm1, Kdm6b
Social Behavior	2.53	6	Tbx1, Nrxn2, Nrxn3, Shank1, Shank2, Cntnap2
Vascular System	2.52	7	Cav3, Myh7, Hcn4, Casq2
Phosphorylation	1.46	68	Myo1a, Myo5b, Smarca4, Tubd1, Mast1, Mast3, Mast4, Met, Dnm3, Fuk

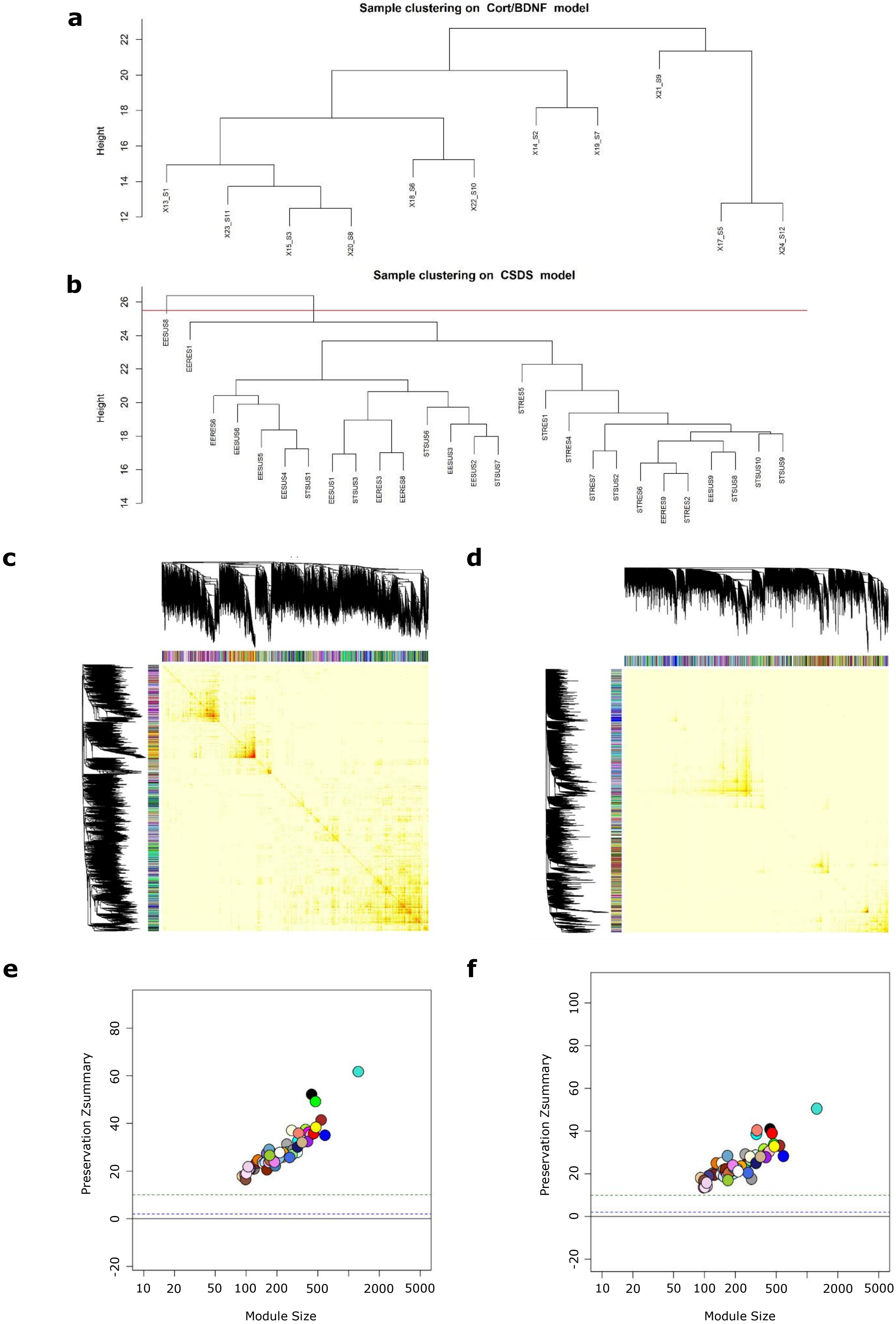

Supplementary Fig. 1

Supplementary Table 3. Differentially expressed genes in the *cyan* and *yellow* modules.

a

Gene Symbol	p-value	log(Fold Change)
Spink8	0.0018	0.5845
Agt	0.003	0.6115
Sparc	0.0051	0.409
Krt73	0.0083	0.4937
Alox12b	0.012	0.4433
Plekhs1	0.0458	0.9487

b

Gene Symbol	p-value	log(Fold Change)
Spink8	0.0002	0.7385
Alg1	0.0009	0.3935
Sema3e	0.0068	0.3917
Sult5a1	0.0077	0.8232
Krt73	0.008	0.4433
Adamts14	0.0102	0.7618
Tph1	0.0118	1.3558
Zap70	0.0122	0.5518
Shisa2	0.0226	0.3845
Gpr139	0.0242	0.4918
Slc9a4	0.0304	0.384
Nhlh2	0.0347	0.4328
Stab2	0.0424	0.5176

c

Gene Symbol	p-value	log(Fold Change)
Adamts14	0.003	0.7126
Cpne7	0.0038	0.578
Alox12b	0.0192	0.4491
Galr2	0.0322	0.4196
Glra1	0.0409	1.133

d

Gene Symbol	p-value	log(Full Change)
Chn2	0.0011	0.6057
Ncf4	0.0022	0.885
Rassf3	0.0031	0.4949
Atp8b1	0.0038	0.7065
Grrp1	0.0046	0.675
Ptprc	0.0052	0.8455
Rasgef1b	0.0054	0.7237
Tlr13	0.0066	0.5613
Gpr153	0.0068	0.5296
Adra1b	0.0098	0.5363
Usp43	0.0147	0.7191
Tnnt1	0.0167	0.8051
Slc5a7	0.0171	1.208
Fcgr1	0.0211	0.6961
Krt12	0.0259	1.1629
Rell1	0.026	0.4882
Medag	0.0266	0.5227
Dysf	0.0294	0.4276
Fign	0.0295	0.5983
Ntsr1	0.0304	0.539
Hs3st5	0.0329	0.6115
Plekhf1	0.033	0.4659
Stil	0.034	0.7658
Cux2	0.0369	0.4095
Cobl	0.0392	0.5536
Gimap6	0.0408	0.3867
Sox7	0.0426	0.4704

e

Gene Symbol	p-value	log(Fold Change)
Socs3	0.0049	0.5483
Kazald1	0.0049	0.3833
Ackr1	0.0122	0.4818
Epha8	0.0198	0.5484
Rgs16	0.0254	0.6734
Foxp2	0.029	0.9195
Chrna6	0.0404	1.3463
Usp43	0.0412	0.7404
Rora	0.0453	0.4583
Plcb4	0.0467	0.4355
Endou	0.0497	0.6653

Supplementary Table 4. Common differentially expressed genes in preclinical models of stress-related disorders and humans with major depressive disorder.

Genes	[1]		[2]			[3]					[4]
vHPC	HPC	AMY	BLA	PFC	NAc	aINS	vmPFC	dlPFC	NAc	vSUB	PFC
Sult5a1		[blue]									[blue]
Zap70											[blue]
Sema3e						[blue]			[blue]		
Chst8									[blue]		
Cpne7					[blue]		[blue]				
Dock9				[blue]							
Gnptab				[blue]							
Sorcs3				[blue]							
Zdbf2				[blue]							
Abca2				[blue]							
Sparc				[blue]							
Kndc1				[blue]							
Akap13				[blue]	[blue]						
Fcgr1			[yellow]								
Gpr88			[yellow]								
Rorb									[yellow]		[yellow]
Sox7								[yellow]			
Rasgrp2	[yellow]									[yellow]	
Prkch	[yellow]										
Hs3st5					[yellow]					[yellow]	
Tnnt1		[yellow]			[yellow]						
Rell1					[yellow]						
Plekhf1					[yellow]						
Cux2						[yellow]	[yellow]			[yellow]	
Rims3							[yellow]			[yellow]	
Chrna4				[yellow]			[yellow]				
Usp43							[yellow]				
Tmsb10							[yellow]				
Meis1							[yellow]				
Kazald1							[yellow]				
Nudt4							[yellow]				
Sowahb										[yellow]	
Cd34										[yellow]	
Trpc3										[yellow]	
Cdh7											[yellow]
Epha8											[yellow]
H2-Q7											[yellow]
Socs3											[yellow]
Ankrd34c											[yellow]
Krt12											[yellow]
Zdhhc22				[yellow]							
Cobl				[yellow]							
Grm4				[yellow]							
Fhod3				[yellow]							
Fign				[yellow]							
Hivep1				[yellow]							
Plcb4				[yellow]							
Foxp2				[yellow]							
Atp8b1				[yellow]							
Deptor				[yellow]							

[1] Bagot RC, Cates HM, Purushothaman I, Vialou V, Heller EA, Yieh L, et al. Ketamine and Imipramine Reverse Transcriptional Signatures of Susceptibility and Induce Resilience-Specific Gene Expression Profiles. Biol Psychiatry. 2017;81(4):285-95.
[2] Paden W, Barko K, Puralewski R, Cahill KM, Huo Z, Shelton MA, et al. Sex differences in adult mood and in stress-induced transcriptional coherence across mesocorticolimbic circuitry. Transl Psychiatry. 2020;10(1):59.
[3] Labonté B, Engmann O, Purushothaman I, Menard C, Wang J, Tan C, et al. Sex-specific transcriptional signatures in human depression. Nat Med. 2017;23(9):1102-11.
[4] Deonaraine KK, Wang Q, Cheng H, Chan KL, Lin H-Y, Liu K, et al. Sex-specific peripheral and central responses to stress-induced depression and treatment in a mouse model. Journal of Neuroscience Research. 2020;98(12):2541-53.

SUPPLEMENTARY LEGENDS

Supplementary Table 1. Body weight over the course of CORT treatment. Body weight was measured one a week during the 6-week treatment. CORT-treated mice showed no difference compared to vehicle-treated mice.

Supplementary Table 2. GO with related genes from RRHO analysis. GO analysis was performed in RRHO quadrants that shared genes with similar biological functions. Quadrants with similar biological networks were aggregated into a "pink" module and a "green" module, as indicated in Figure 2. Columns represent the (i) gene ontology term, (ii) enrichment score and (iii) total number of genes obtained from DAVID annotation clustering; (iv) genes selected based on their known role in neuronal function. **Supplementary Figure 1. Validation of Consensus Coexpression Network.** Hierarchal clustering analysis for a) the CORT model and b) the chronic social defeat stress (CSDS) model. Modules with high mutual similarity of greater than 85% were merged. Topological overlap matrix (TOM) of WGCNA modules for c) the CORT model and d) the CSDS model. Increasing color intensity from white to dark red within the TOM indicates increasing coexpression-based topological overlap. Preservation Z-summary of network modules as compared to their size in genes for e) the CORT model and f) the CSDS model. Modules with preservation Z-scores greater than 10 (above the dotted green line) and not less than 2 (below the dotted blue line) were indicated to be strongly preserved in the data set.

Supplementary Table 3. Differentially expressed genes in the *cyan* and *yellow* modules. The tables depicts differentially expressed hub genes of the *cyan* (a-c) and *yellow* (d,e) module in a) WT mice under CORT, b) BDNF het-Met mice under CORT, c) SUS mic in SH, d) BDNF het-Met mice under vehicle, and e) SUS mice in EE. Rows are highlighted in cyan or yellow to indicate gene hubs that are shared across groups.

Supplementary Table 4. Common differentially expressed genes in preclinical models of stress-related disorders and humans with major depressive disorder. The table depicts shared differentially expressed genes between the gene hubs of the *yellow* or *cyan* module and datasets from models that use different stressors or humans with major depressive disorder that investigate genomic signatures of stress coping in multiple brain regions. Cells are highlighted in either yellow or cyan depending on the module that overlaps with the gene of interest.
